# Radial Nerve Palsy Associated with Humeral Shaft Fractures in Children

**DOI:** 10.1155/2023/3974604

**Published:** 2023-12-01

**Authors:** Wiktor Łukasz, Tomaszewski Ryszard, Damps Maria

**Affiliations:** ^1^Department of Trauma and Orthopaedic Surgery, Upper Silesian Children's Health Centre, Katowice, Poland; ^2^Department of Trauma and Orthopedic Surgery, ZSM Hospital, Chorzów, Poland; ^3^Faculty of Science and Technology, Institute of Biomedical Engineering, University of Silesia in Katowice, Katowice, Poland; ^4^Department of Anaesthesiology and Intensive Care, Upper Silesian Child Health Centre, Faculty of Medical Sciences in Katowice, Medical University of Silesia, Katowice, Poland

## Abstract

**Background:**

This is the first systematic review of the relationship between humeral shaft fractures and radial nerve palsy in children. The present comprehensive review is aimed at identifying important clinical findings between humeral diaphysis fractures and radial nerve injuries and assessing the effects of treatment.

**Methods:**

We searched electronic bibliographic databases, including PubMed, the Cochrane Library, Scopus, and Web of Knowledge, until March 2022. This systematic review was performed according to the Preferred Reporting Items for Systematic Reviews and Meta-Analyses and the patients, interventions, comparisons, outcomes guidelines.

**Results:**

We identified 23 original papers, of which 10 were eligible for further analysis. Cases of 32 young patients with radial nerve palsy were identified and analyzed. The prevalence of radial nerve palsy was 4.34% (eight cases out of 184 patients with humeral shaft fractures). The radial nerve was most often associated with a simple transverse fracture (12A3, 17 cases (65.4%)).

**Conclusions:**

Radial nerve injury in humeral shaft fractures in children is rare, with a frequency of 4.34%. We highly recommend early surgical nerve exploration with transverse fractures in the distal third segment combined with primary radial palsy. Furthermore, we recommend making thoughtful decisions regarding early nerve exploration in the Holstein–Lewis fractures. In addition, consideration of early surgical nerve exploration in fractures resulting from high-energy trauma and open fractures despite their morphology is recommended.

## 1. Introduction

Humeral shaft fractures are quite rare in the pediatric population, constituting 0.4% to 3% of pediatric fractures and 10% of humerus fractures [1, 2]. Meanwhile, humeral supracondylar fractures are among the most common fractures in children, constituting around 15% of all pediatric fractures [3]. The radial nerve, owing to its close proximity to the bone, is at a high risk of being damaged [4]. Radial nerve injury is the most common nerve complication among long bone fractures, with a prevalence of 7% to 17% in adults [5–10]. However, owing to the rarity of this condition in skeletally immature patients, radial palsy caused by humeral diaphysis fractures is still not comprehensively described. There is no valid algorithm to guide surgeons in the management of the condition. Studies on adults have shown that such cases are complex medical problems, and no systematic reviews have included children. Because a comprehensive review may identify important clinical findings, we aimed to provide a systematic review of studies on this problem.

## 2. Material and Methods

### 2.1. Search Strategy and Study Acquisition

**Figure 1:**
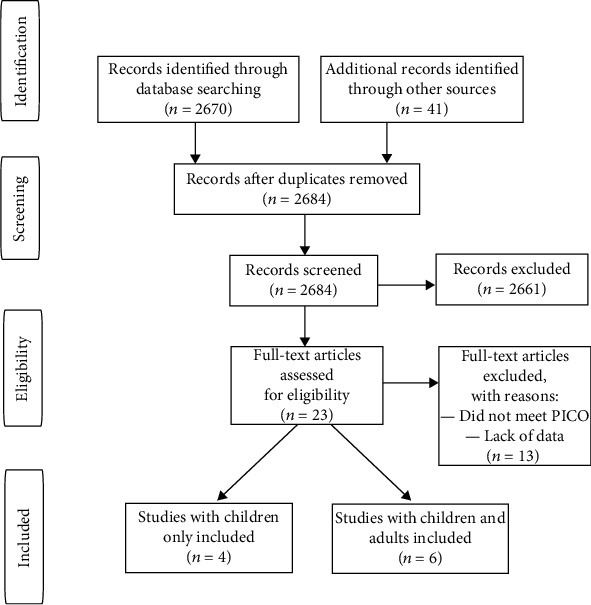
PRISMA protocol for data acquisition.

This systematic review was performed according to the patients, interventions, comparisons, outcomes (PICO) and Preferred Reporting Items for Systematic Reviews and Meta-Analysis guidelines. PubMed, the Cochrane Library, Scopus, and Web of Knowledge databases were searched. Search queries used include “humeral shaft fracture” or “humeral diaphysis fracture”, “children” or “immature” and “radial nerve palsy” or “radial nerve paralysis”. No filter was used. Two authors screened the results. The initial search yielded 2,711 results. After the preliminary revision, 23 papers were selected for further analysis. Ten papers fulfilled the inclusion criteria and were included in this study. The study inclusion data is presented in Figure 1.

### 2.2. Inclusion and Exclusion Criteria

We used PICO framework-based research questions for this review. The PICO criteria used in this study are listed in Table 1. Articles that met the predefined criteria were included. The criteria for paper eligibility were as follows: it was published in English, included patients younger than 18 years with humeral shaft fracture-radial nerve palsy, and extracted data focused on the decision-making and treatment of pediatric patients. Articles were excluded if they were not original, the full text was not available, were not written in English, or did not conform to the PICO guidelines.

### 2.3. Data Extraction

**Figure 2:**
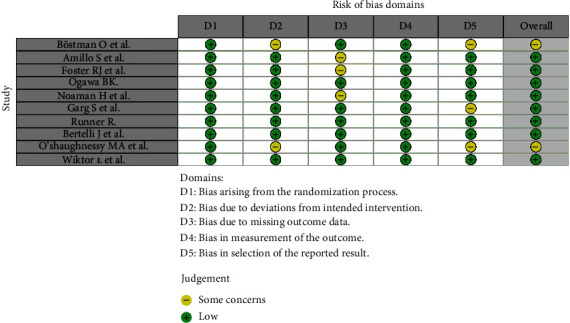
“Traffic light” plots of the domain-level judgements for each individual result.

**Figure 3:**
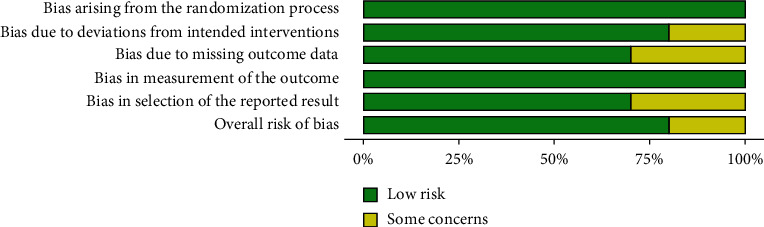
Weighted bar plots of the distribution of risk-of-bias judgements within each bias domain.

Patient data, including age, sex, humeral fracture type, nature of radial palsy, method of fracture treatment, method of damaged nerve treatment, and its final effect, were carefully extracted. To reduce bias, each parameter was carefully defined and double-checked by two authors using the Robvis Cochrane tool/ROB2 (Figures 2 and 3) [11].

The Coleman Methodology Score was used to assess the quality of the studies. The study methodology was assessed based on 10 criteria, giving a total score between 0 and 100 (Table 2). A score of 100 indicated that the study largely avoided chance, various biases, and confounding factors.

## 3. Results

**Figure 4:**
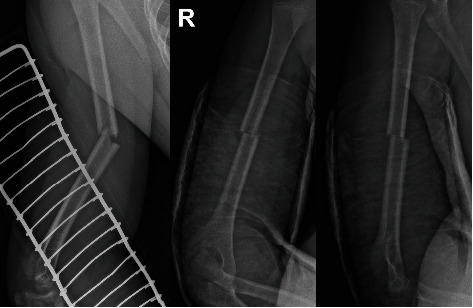
X-ray of the simple transverse humeral shaft fracture (12A3b), most often associated with radial nerve palsy in children (figures from the Department of Trauma and Orthopedic Surgery database, Upper Silesian Children's Health Center, John Paul, Katowice, Poland).

**Figure 5:**
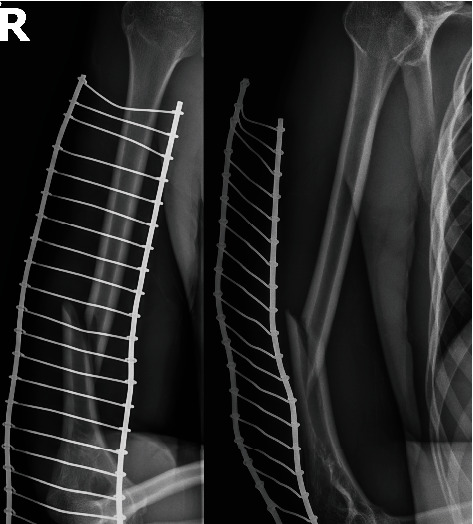
X-ray of the spiral humeral shaft fracture at distal third (12A1c-Holstein–Lewis fracture) usually not associated with severe radial nerve damage in children (figures from the Department of Trauma and Orthopedic Surgery database, Upper Silesian Children's Health Center, John Paul, Katowice, Poland).

We identified 23 original papers, of which 10 were eligible for further analysis. Thirteen studies were excluded because of a lack of crucial data regarding pediatric patients. A list of the excluded studies is presented in Table 3. Details of the included studies are presented in Table 4. Four studies exclusively included children, whereas the remaining studies described adults and children. We identified eight retrospective studies and two case reports. From the 10 included studies, 32 skeletally immature patients with radial nerve palsy were identified, and their data were analyzed. Based on studies describing results of humeral shaft fractures in children, the overall prevalence of radial nerve palsy was 4.34% (8/184 patients with humeral shaft fractures). Only one study favored early exploration in radial palsy cases; in other studies, decision-making processes were chosen individually with no strict guidelines. The humeral shaft was divided into three parts: no nerve damage was found in the proximal third, 12 radial palsies in the middle third (37.5%), and 18 palsies in the distal third (56.25%). In two cases, the precise fracture level was not defined. Twenty-six fractures were classified based on the Müller AO classification of fractures scale. Damage to the radial nerve was mostly associated with simple transverse fracture (12A3, 17 cases (65.4%)); simple transverse fracture occurred in the middle third (12A3b) in ten cases (38.46%), as shown in Figure 4, and in the distal third (12A3c) in seven cases (26.92%). We identified six simple spiral fractures (12A1), five of which were related to the distal third (12A1c, 19.23%), as shown in Figure 5, and one to the middle third (12A1b, 3.84%). Moreover, radial palsy occurred in two oblique fractures located in the distal humeral third (12A2c, 7.69%) and in one intact wedge fracture located in the distal third (12B2c, 3.84%). In 25 cases, the symptoms of radial nerve damage appeared immediately after a humeral fracture (primary palsy), and symptoms were delayed in seven cases (secondary palsy). In 15 (46.87%) cases, radial palsy was related to a humeral fracture resulting from high-energy trauma; however, in 15 cases, the mechanism of the injury was not clearly defined. There were six open and 29 closed humeral fractures. The most common radial nerve injury was neuropraxia (11, 34.37%), followed by neurotmesis (7, 21.87%) and nerve entrapment (7, 21.87%), as shown in Table 5. Expectant observations were made in 10 patients with symptoms of radial nerve injury. Spontaneous recovery was observed in all the patients. Primary operative treatment related to the nerve procedure was defined as an intervention within 2 weeks after injury. Early nerve exploration was performed in 12 patients, and late nerve exploration was performed in 10 patients (more than 14 days after the onset of nerve injury symptoms).

## 4. Discussion

Humeral shaft fractures are quite rare in the pediatric population, with an overall prevalence of 0.4% to 3% of all pediatric fractures [1, 2]. The frequency of radial nerve injury in humeral shaft fractures in adults ranges from 7% to 17% [5–10]. Based on studies describing the results of humeral shaft fractures in children included in our review [32, 33], the overall prevalence of radial nerve palsy was 4.34% (8/184 patients with humeral shaft fracture).

Based on these data, the incidence of radial nerve injury in children is much lower than that in adults and is similar to that reported by other authors' studies [34, 35]. In the present review, radial nerve injury was mostly associated with simple transverse fracture (12A3, 17 cases, 65.4%), consistent with findings in the literature [7, 36, 37]. Ten of these were in the middle third (12A3b, 38.46%), and seven were in the distal third (12A3c, 26.92%). Six of the 10 simple transverse fractures in the middle third were neuropraxia, with spontaneous recovery after expectant observation. Two patients underwent early nerve exploration and neurolysis with full radial nerve recovery (one nerve was compressed and one stretched). Two cases of neurotmesis were reported, one of which was treated with delayed neurorrhaphy 92 days after the initial injury, and the other with radial nerve reconstruction using sural cable grafts. Functional recovery was observed in both cases. Out of seven simple transverse fractures in the distal third, only one case of neuropraxia was followed up, and there was spontaneous recovery after expectant observation. In two cases, early surgical neurolysis was performed with radial nerve recovery (one bruised nerve and one entrapped nerve). Two patients required nerve reconstruction with sural cable grafts, with good functional outcomes (one nerve entrapped and one lacerated). There were two cases of early neurorrhaphy: one resulted in the restoration of nerve function, and the other (open humeral fracture IIIB (according to GA)) required secondary tendon transfers due to the lack of nerve function. The analysis of fracture types identified five simple spiral fractures related to the distal humeral third (12A1c, Holstein–Lewis fracture, 19.23%). There were five spiral humeral fractures. Of these, three were neuropraxic, including two cases after expectant observation and one after unnecessary surgical exploration (intact radial nerve) that resulted in good outcomes. The other two cases underwent early surgical exploration and neurolysis of the compressed nerve, combined with fracture fixation using a dynamic compression plate. Nerve recovery was observed in these patients. Contrary to the reports by Holstein and Lewis and Ekholm et al. [38, 39], we found no serious damage to the radial nerve in patients with spiral fractures of the distal third. These observations are in line with the reports of Whitson, which stated that the radial nerve at the middle third of the humeral shaft was quite mobile and there was a soft tissue protective layer between the nerve and the humerus; therefore, radial nerve damage at this level is generally due to neuropraxia and has a high potential for spontaneous recovery [40]. The distal third radial nerve passes through the lateral intermuscular septum and stays close to the humeral diaphysis; therefore, nerve damage caused by fracture is caused by contusion, entrapment, or laceration [40]. The risk of radial nerve damage is high in patients with open humeral fractures [5–7, 14, 41]. Four of the six cases with open humeral shaft fractures included in the review had a radial nerve injury requiring surgical repair (two patients with neurorrhaphy and two patients with radial nerve reconstruction). In such cases, early surgical exploration is justified, as it offers the possibility of direct radial nerve repair. In one of the analyzed cases with an open fracture (type II according to the Gustilo-Anderson open fracture classification scale), repair of the damaged nerve was postponed until the fracture and soft tissues healed due to significant wound contamination [33]. The patient regained functional recovery 15.5 months after the initial injury. The crucial question is whether to treat radial nerve palsy in close humeral shaft fractures conservatively or surgically and whether the patient may benefit from early nerve exploration. We could not perform any effective analysis of the treatment methods. The valid standard of clinical decision-making should be based on direct comparison, which was not possible in our review because of the heterogeneity of patients. The small group of patients was also a significant problem. Noaman et al. recommended early operative treatment (within the first 2 weeks) in cases of open humeral fractures with radial nerve injury; fractures of the distal third of the humerus, either transverse or oblique; radial nerve injury in Holstein–Lewis fractures; and postreduction radial nerve injury [28]. Böstman et al. supported observing expectations in both immediate and secondary radial nerve palsies. In their opinion, early exploration could be considered in fractures showing a bayonet position between fragments, which would result in abundant callus formation, thereby endangering the recovery of the radial nerve [24]. Amillo et al. recommended early surgical exploration in open fractures, when open reduction and internal fixation are necessary, in fractures associated with vascular injury, and in cases with palsy after close reduction of a fracture. In open fractures related to neurotmesis, they suggest grafting with sural nerve cable grafts. Late nerve exploration was proposed if there were no clinical or electrophysiological signs of nerve recovery after three months [25]. O'Shaughnessy et al. suggested that expectant observation is a safe course for pediatric humeral shaft fractures; however, their study was based on three cases of neuropraxia associated with closed humeral fractures [32]. They referred to a meta-analysis by Shao et al., which found a 4.3-month average wait period before surgical exploration in the adult population [6]. Wiktor and Tomaszewski preferred expectant observation without nerve exploration in fractures caused by low-energy trauma. However, early surgical nerve exploration related to fracture stabilization is recommended in fractures after high-energy trauma [33, 42]. Moreover, they declared that early exploration during fracture repair is safer than delayed exploration and further reduces nerve damage after fracture stabilization.

The main limitation is that the vast majority of the literature has focused on radial nerve palsy in humeral shaft fractures in adult patients, some of whom include children. There are only a few papers that include children; therefore, a systematic review is very difficult.

### 4.1. Limitations

This study has some limitations. The main limiting factors of the present study were the low number of patients and the heterogeneity of the included studies. These limitations occurred because of the limited number of studies available in the literature. This makes it difficult to draw definitive conclusions.

### 4.2. Strengths

The main strength of this study was its novelty. To our knowledge, this is the first systematic review of radial nerve palsy associated with humeral shaft fractures in children. It systematically summarizes the clinical findings on this rare topic.

## 5. Conclusions

Radial nerve injury in humeral shaft fractures in children is rare, with a frequency of 4.34%. The incidence is significantly lower than that in adults. Radial nerve palsy is often associated a with simple transverse humeral shaft fracture (12A3). There is a significant difference in the nature of radial nerve injury between transverse fractures depending on the fracture level. Radial palsy with fractures in the middle third often takes the form of neuropraxia with spontaneous recovery, whereas radial injury with distal third fractures usually requires surgical nerve exploration. Based on this, we highly recommend the consideration of early surgical nerve exploration for transverse fractures in the distal third combined with primary radial palsy. Spiral fractures of the distal third 12A1c (Holstein–Lewis fracture) are usually not associated with severe radial nerve damage; therefore, we recommend making thoughtful decisions about early nerve exploration. Although the morphology of fractures resulting from high-energy trauma and that of open fractures are associated with a high risk of serious nerve damage, we highly recommend the consideration of early surgical nerve exploration.

## Figures and Tables

**Table 1:** PICO criteria used in the study.

PICO	Description
Patients	Patients under 18 years with humeral shaft fracture complicated with radial nerve palsy
Intervention	Expectant observation or surgical exploration
Comparisons	The frequency and nature of radial nerve injury depending on the level and morphology of humeral diaphysis fracture
Outcomes	The possible outcomes depending on different treatment approaches

**Table 2:** Modified Coleman Methodology Score.

Part A: only one score to be given for each of the 7 sections	
1. Study size: number of patients	
<30	0
30-50	4
51-100	7
>100	10
2. Mean follow-up	
<12 mo	0
12-36 mo	4
37-60 mo	7
>61 mo	10
3. Surgical approach	
Different approaches used and outcomes not reported separately	0
Different approaches used and outcomes reported separately	7
Single approach used	10
4. Type of study	0
Retrospective cohort study	5
Prospective cohort study	10
Randomized controlled trial	
5. Description of diagnosis	
Described without percentage specified	0
Described with percentage specified	5
6. Descriptions of surgical technique	
Inadequate (not stated, unclear)	0
Fair (technique only stated)	5
Adequate (technique stated, details of surgical procedure given)	10
7. Description of postoperative rehabilitation	
Described	5
Not described	0

Part B: scores may be given for each option in each of the 3 sections if applicable	
1. Outcome criteria	2
Outcome measures clearly defined	2
Timing of outcome assessment clearly stated	3
Use of outcome criteria that has reported reliability	3
General health measure included	
2. Procedure of assessing outcomes	
Participants recruited	5
Investigator independent of surgeon	4
Written assessment	3
Completion of assessment by patients themselves with minimal investigator assistance	33
3. Description of subject selection process	
Selection criteria reported and unbiased	5
Recruitment rate reported	
>90%	5
<90%	0

**Table 3:** List of excluded studies.

Author (date of publication)	No	Age	Proximal (P); middle (M); distal (D) third; segmental (S)	Open/close	Radial palsy/children
Duthie (1957) [12]	1	15	1 M-D junction	Close	1
Shaw and Sakellarides (1967) [13]	45	6-71 (NA)	3 P; 42 M-D	8/37	9
Packer et al. (1972) [14]	31	5-88 (52)	16 M; 14 D; 1 S	0/31	NA
Pollock et al. (1981) [15]	24	0-62 (38)	2 P; 5 M; 14 D; 3S	3/21	6: 0-10 yo (3); 10-20 yo (3)
Kaiser et al. (1981) [16]	54	5-80 (NA)	37 M; 4 M-D junction; 13 D	6/49	NA
Shah and Bhatti (1983) [17]	62	1-80 (NA)	4 P; 25 M; 33 D	15/47	16: 0-10 yo (5); 11-20 yo (11)
Sonneveld et al. (1987) [18]	17	3-74 (43)	16 M	1/16	NA
Postacchini and Morace (1988) [19]	42	5-73 (29)	1 PM junction; 11 M; 19 M-D junction; 11 supracondylar	7/35	7
Ogawa and Yoshida (1998) [20]	14	12-43 (25)	14 D	0/14	NA
Larsen and Barfred (2000) [8]	26	9-79 (21)	NA	0/26	NA
Kim et al. (2001) [21]	57	8-64 (42)	NA	NA	NA
Lim et al. (2001) [22]	23	0-60 (NA)	13 M; 10 D	6/17	NA
Hoa et al. (2017) [23]	26	36 (7-83)	1 P; 11 M; 8 D	5/21	NA

NA: not available.

**Table 4:** The details of included studies.

Author (article type)	Publishing year	Study period	Patients included in the review from original study	Male/female	Age/year	Nerve damage type	Radial palsy	Intervention type	Mean time from injury to surgery	M-middle; D-distal third	Close/open fracture	Injury mechanism	Fracture type AO classification	Coleman Methodology Score
Böstman et al. (retrospective study) [24]	1986	1961-1980	10 out of 75 radial palsy cases	8/2	13,7 (6-16)	Primary-6; secondary-4	Neuropraxia-6; bruising-1; laceration-2; intact-1	Expectant observation-6; exploration-4 (neurorrhaphy in 2 cases)	23,75 days (1-92)	M-6; D-4	7/3	NA	12A1-2; 12A2-1; 12A3-7	61
Amillo et al. (retrospective study) [25]	1993	1972-1988	4 out of 12 radial palsy cases	3/1	9,25 (4-18)	Primary-4	Divided-1; entrapped-1; fibrosis-2	neurolysis-2; tendon transfer-1; grafting-1 (3 cable grafts of sural nerve)	5,75 months (2-15)	M-1; D-3	4/0	NA	NA	65
Foster et al. (retrospective study) [26]	1993	1981-1992	1 out of 14 radial palsy cases	1/0	17	Primary	Laceration-1	Epineural neurorrhaphy-1	NA	NA	0/1	Motor vehicle accident	NA	55
Ogawa et al. (case report) [27]	2007	2007	1 case	0/1	3	Primary	Neurotmesis-1	Grafting (3 cable grafts of sural nerve)	3 months	M-1	1/0	Motor vehicle accident	12A3	58
Noaman et al. (retrospective study) [28]	2008	2001-2007	4 out of 36 radial palsy cases	2/2	14,25	Primary-3; secondary-1	Entrapped-1; compressed-3	Early exploration-4	5,7 months (3-14)	M-1; D-3	4/0	Car accident-3; fall from height-1	12A1-2; 12A3-2	63
Garg et al. (retrospective study) [29]	2009	1999-2006	2 out of 13 shaft fracture cases	0/2	12 (9,1-16,7)	Primary-2	Neuropraxia-1; neurotmesis-1	Neurolysis-1; neurorrhaphy-1 and tendon transfer because of failure	NA	D-1; other-NA	0/2	Motor vehicle-1; terrain vehicle-1 accident	12A3-1; NA	51
Runner and Whicker (case report) [30]	2017	2017	1 case	1/0	12	Secondary (4 days after reposition)	Stretched over sharp bone fragment	Neurolysis-1	11 days	M-1	1/0	Motor vehicle accident	12A3	58
Bertelli et al. (retrospective study) [31]	2018	1997-2015	1 out of 7 radial palsy cases	NA	9	Primary	Entrapped-1	Grafting (3 cable grafts of sural nerve)	6 months	D-1	0/1	NA	12A3	55
O'Shaughnessy et al. (retrospective study) [32]	2019	1996-2016	3 out of 80 shaft fracture cases	NA	10	Primary-3	Neuropraxia-3 (4% of all humeral shaft fracture)	Expectant observation-3	0	M-2; D-1	3/0	Motor vehicle accident	12A1-1; 12A3-2	71
Wiktor and Tomaszewski (retrospective study) [33]	2022	2011-2021	5 out of 104 shaft fracture cases	4/1	13,6 (8,6 -17,2	Primary-4; secondary-1	Neuropraxia-1; neurotmesis-2; entrapped-2	Expectant observation-1; neurolysis-2; grafting-2 (3 cable grafts of sural nerve)	44,75 days (0-140)	M-1; D-4	3/2	Fall from height-2; hit by car-1; low energy-2	12A1-1; 12A2-1; 12A3-2; 12B2 -1	70

NA: not available.

**Table 5:** Particular list of patients.

	Sex/age	Fracture classification	Trauma	Nerve damage	Surgical treatment	Outcome	Detailed outcome
1.1	Boy/14 years	Close/simple transverse/middle thirds 12A3b	NA	Secondary/neuropraxia	Expectant observation	Recovery	

1.2	Boy/15 years	Close/simple spiral/distal thirds 12A1c	NA	Primary/neuropraxia	Expectant observation	Recovery	

1.3	Boy/16 years	Close/simple transverse/distal thirds 12A3c	NA	Primary/neuropraxia	Expectant observation	Recovery	

1.4	Boy/6 years	Close/simple transverse/middle thirds 12A3b	NA	Secondary/neuropraxia	Expectant observation	Recovery	

1.5	Girl/13 years	Close/simple transverse/middle thirds 12A3b	NA	Primary/neuropraxia	Expectant observation	Recovery	

1.6	Boy/14 years	Close/simple transverse/middle thirds 12A3b	NA	Primary/neuropraxia	Expectant observation	Recovery	

1.7	Boy/13 years	Close/simple transverse/middle thirds 12A3b	NA	Secondary/bruising	Nerve exploration with neurolysis 1 day after injury	Recovery	

1.8	Boy/16 years	Close/simple transverse/middle thirds 12A3b	NA	Primary/neurotmesis	Nerve exploration with neurorrhaphy 92 days after injury	Recovery	

1.9	Boy/15 years	Close/simple oblique/distal thirds 12A2c	NA	Primary/neurotmesis	Nerve exploration with neurorrhaphy 1 day after injury	No recovery	

1.10	Girl/15 years	Close/simple spiral/distal thirds 12A1c	NA	Secondary/intact	Nerve exploration 1 day after injury	Recovery	

2.1	Boy/11 years	Close/distal thirds NA	NA	Primary/neurotmesis	CRIF/K-wires nerve surgery 15 months after injury (nerve lost >10 cm)	No recovery	Wrist M0 Fingers M0 Thumb M0

2.2	Boy/4 years	Close/distal thirds NA	NA	Primary/callus entrapment	CRIF/K-wires nerve reconstruction with a sural nerve cable graft (3×) 2 months after injury	Recovery after 15 months after injury	Wrist M5 Fingers M4 Thumb M4

2.3	Girl/18 years	Close/middle thirds NA	NA	Primary/fibrosis	CRIF/Hackethal nail Late nerve exploration with neurolysis 3 months after injury	Recovery after 24 months after injury	Wrist M4 Fingers M4 Thumb M3

2.4	Boy/4 years	Close/distal thirds NA	NA	Primary/fibrosis	CRIF/Hackethal nail Late nerve exploration with neurolysis 3 months after injury	Recovery 12 months after injury	Wrist M5 Fingers M5 Thumb M5

3.1	Boy/17 years	Open IIIA wg GA NA	High-energy trauma/motor vehicle accident	Primary/laceration	ORIF/compression plate + nerve exploration with neurorrhaphy	Recovery	Wrist M5 Fingers M5 Thumb M5

4.1	Girl/3 years	Close/simple transverse/middle thirds 12A3b	High-energy trauma/motor vehicle accident	Primary/neurotmesis	Nerve reconstruction with a sural nerve cable graft (3×) 3 months after injury	Recovery 6 months after surgery	Wrist M5 Fingers M5 Thumb M5

5.1	Boy/8 years	Close/simple transverse/distal thirds 12A3c	High-energy trauma/car accident	Secondary/entrapment between bone fragments	ORIF/DCP plate + early nerve exploration with neurorrhaphy	Recovery	Excellent motor function

5.2	Girl/16 years	Close/simple spiral/distal thirds 12A1c	High-energy trauma/car accident	Primary/compression	ORIF/DCP plate + early nerve exploration with neurolysis	Recovery	Excellent motor function

5.3	Girl/17 years	Close/simple transverse/distal thirds 12A3b	High-energy trauma/fall from hight-1	Primary/compression	ORIF/DCP plate + early nerve exploration with neurolysis	Recovery	Excellent motor function

5.4	Boy/16 years	Close/simple spiral/distal thirds 12A1c	High-energy trauma/car accident	Primary/compression	ORIF/DCP plate + early nerve exploration with neurolysis	Recovery	Excellent motor function

6.1	Girl/10.2 years	Open IIIA wg GA NA	High-energy trauma/terrain vehicle accident	Primary/neuropraxia	ORIF/FIN Neurolysis	Spontaneous recovery after 4 months	

6.2	Girl/9.1 years	Open IIIB wg GA Simple transverse/distal thirds 12A3c	High-energy trauma/motor vehicle accident	Primary/neurotmesis	ORIF/FIN + neurorrhaphy. -1 and tendon transfer because of failure	Tendon transfer because of failure 12 months after injury	Wrist M0 Fingers M0 Thumb M0

7.1	Boy/12 years	Close/simple transverse/middle thirds 12A3b	High-energy trauma/motor vehicle accident	Secondary (4 days after reposition)/stretched over sharp bone fragment	Nerve exploration with neurolysis 16 days after injury.	Recovery after 6 weeks	Wrist M5 Fingers M5 Thumb M5

8.1	9 years	Open/simple transverse/distal thirds 12A3c	NA	Primary/entrapment between bone fragments	Nerve reconstruction with a sural nerve cable graft (3×) 6 months after injury.	Recovery	Wrist M4 Fingers M4 Thumb M4

9.1		Close/simple spiral/distal thirds 12A1c	High-energy trauma/high-speed dirt bike	Primary/neuropraxia	Nonoperative treatment/expectant observation	Spontaneous recovery after 3 weeks	

9.2		Close/simple transverse/middle thirds 12A3b	High-energy trauma/motor vehicle accident	Primary/neuropraxia	Nonoperative treatment/expectant observation	Spontaneous recovery	

9.3		Close/simple transverse/middle thirds 12A3b	High-energy trauma/motor vehicle accident	Primary/neuropraxia	Nonoperative treatment/expectant observation	Spontaneous recovery	

10.1	Boy/8.6 years	Close/simple spiral/middle thirds AO 12A1b	Low-energy trauma	Primary/neuropraxia	CRIF/FIN Expectant observation	Spontaneous recovery after 2.5 months	

10.2	Girl/16.3 years	Close/simple oblique/distal thirds AO 12A2c	High-energy trauma/fall from 8 meters	Primary/neurotmesis	ORIF/titan plate + nerve reconstruction with a sural nerve cable graft (3×)	Recovery after 7.5 months	Wrist M4 Fingers M4 Thumb M4

10.3	Boy/13.5 years	Close/simple transverse/distal thirds AO 12A3c	Low-energy trauma	Secondary/entrapment between bone fragments	ORIF/titan plate + nerve exploration 23 days after injury – 2 days after nerve palsy	Recovery after 3.3 months	Wrist M4 Fingers M4 Thumb M4

10.4	Boy/17.2 years	Open GA 1/intact wedge/distal thirds AO 12B2c	High-energy trauma/fall from 6 meters	Primary/entrapment between bone fragments	CRIF/external fixator ORIF/titan plate + nerve exploration 16 days after injury	Recovery after 4.6 months	Wrist M4 Fingers M4 Thumb M4

10.5	Boy/12.3 years	Open GA 2/simple transverse/distal thirds AO 12A3c	High-energy trauma/hit by a car	Primary/neurotmesis	ORIF/FIN With no nerve exploration due to wound contamination Implant removal + nerve reconstruction with a sural nerve cable graft (3×) 4.5 months after injury	Recovery 5.5 months after nerve reconstruction/15.5 months after injury!	Wrist M4 Fingers M4 Thumb M4

NA: not available; CRIF: close reduction and internal fixation; ORIF: open reduction and internal fixation; FIN: flexible intramedullary nailing; DCP: dynamic compression plate; BMRC: British Medical Research Council Rating Scale; GA: Gustilo-Anderson open fracture classification scale.

## Data Availability

All data is available at the Department of Children's Orthopedics, John Paul II Upper Silesian Child Health Center, Katowice, Poland. Access to data is possible with permission from the responsible author.

## Abbreviations

PICO: Patients, interventions, comparisons, outcomes
PRISMA: Preferred Reporting Items for Systematic Reviews and Meta-Analyses
CRIF: Close reduction and internal fixation
ORIF: Open reduction and internal fixation
FIN: Flexible intramedullary nailing
DCP: Dynamic compression plate
BMRC: British Medical Research Council Rating Scale
GA: Gustilo-Anderson open fracture classification scale.
